# Perceived Parental Distraction by Technology and Mental Health Among Emerging Adolescents

**DOI:** 10.1001/jamanetworkopen.2024.28261

**Published:** 2024-08-16

**Authors:** Audrey-Ann Deneault, André Plamondon, Ross D. Neville, Rachel Eirich, Brae Anne McArthur, Suzanne Tough, Sheri Madigan

**Affiliations:** 1 Département de Psychologie, Université de Montréal, Montréal, Quebec, Canada; 2 Faculté des Sciences de L’Éducation, Université Laval, Quebec City, Québec, Canada; 3 School of Public Health, Physiotherapy and Sports Science, University College Dublin, Dublin, Ireland; 4 Department of Psychology, University of Calgary, Calgary, Alberta, Canada; 5 Alberta Children’s Hospital Research Institute, Calgary, Canada; 6 Department of Community Health Sciences, Department of Pediatrics, University of Calgary, Calgary, Alberta, Canada

## Abstract

**Importance:**

The digital phenomenon termed *technoference* refers to interruptions in routine social interactions due to technology use. Technoference may negatively affect parents’ attention to cues necessary for supporting children’s mental health.

**Objective:**

To explore whether there are directional prospective associations between perceived parental technoference and emerging adolescents’ mental health symptoms (anxiety, depression, inattention, and hyperactivity).

**Design, Setting, and Participants:**

This cohort study assessed a general population of mothers and emerging adolescents in Calgary, Alberta, Canada. Women were recruited during pregnancy between May 3, 2008, and December 13, 2010, with convenience sampling and repeated follow-up; eligible women were 18 years or older, spoke English, had a gestational age of at least 24 weeks, and received local prenatal care. Data collection for the present study took place when emerging adolescents were aged 9 (May 20 to July 15, 2020), 10 (March 4 to April 30, 2021), and 11 (November 22, 2021, to January 17, 2022) years. Mothers provided consent for their child to participate, and emerging adolescents provided assent. Data were analyzed from December 1 to 31, 2023, using random-intercept cross-lagged panel models.

**Exposure:**

Perceived parental technoference.

**Main Outcomes and Measures:**

Emerging adolescents completed questionnaires about their perception of parental technoference and their mental health symptoms (depression, anxiety, hyperactivity, and inattention) at the 3 study times. This study did not rely on statistical significance, but instead on the magnitude of effect sizes to determine meaningful effects.

**Results:**

Participants included 1303 emerging adolescents (mean [SD] age, 9.7 [0.8] years at time 1; of the 1028 reporting information, 529 [51.5%] were girls). Cross-sectional associations indicated correlations between perceptions of parental technoference and emerging adolescents’ mental health (*r* range, 0.17-0.19). Higher levels of anxiety at 9 and 10 years of age were prospectively associated with higher parental technoference scores at 10 (β = 0.11 [95% CI, −0.05 to 0.26]) and 11 (β = 0.12 [95% CI, 0.001-0.24]) years of age, with small magnitudes of effect size. Higher parental technoference scores at 9 and 10 years of age were prospectively associated with higher hyperactivity at 10 (β = 0.07 [95% CI, −0.07 to 0.22]) and 11 (β = 0.11 [95% CI, −0.02 to 0.24]) years of age and inattention at 11 years of age (β = 0.12 [95% CI, 0.001-0.24]), with small magnitudes of effect size. No gender differences were identified.

**Conclusions and Relevance:**

In this 3-wave longitudinal birth cohort study, perceived parental technoference was associated with emerging adolescents’ mental health. The findings speak to the need to discuss digital technology use and mental health with parents and emerging adolescents as a part of routine care.

## Key Points

**Question:**

What are the longitudinal associations between perceived parental digital interruptions (technoference) and mental health difficulties in emerging adolescents?

**Findings:**

In a cohort study of 1303 emerging adolescents aged 9 to 11 years across 3 assessments, higher levels of anxiety symptoms were associated with higher levels of perceived parental technoference later in development. Higher levels of perceived parental technoference were associated with higher levels of inattention and hyperactivity symptoms later in development.

**Meaning:**

Parent digital technology use that interrupts routine parent-adolescent interactions may be instigated by the emerging adolescents’ levels of anxiety, and parent technoference may also have consequences for emerging adolescents’ inattention and hyperactivity.

## Teaser

This cohort study examines the bidirectional associations between emerging adolescents’ perceptions of parental digital interruptions (technoference) and their anxiety, depression, attention difficulties, and hyperactivity symptoms.

## Introduction

Digital technology is woven into the fabric of modern family life.^1^ Smartphones, tablets, and other digital devices help families with communication, scheduling, and entertainment. Despite its benefits, routine technology use (eg, texting, scrolling through social media) can also disrupt interactions between parents and their children of all ages, a phenomenon encapsulated by the understudied concept of *technoference*.^2^ A recent phone-tracking study of parents with young infants found that parents spend 5.12 hours per day on their smartphones and 27% of the time with their infant engaged with their digital device.^3^ Similar rates are identified across age groups, with 68% of US parents with a child younger than 17 years reporting that they become distracted by their smartphones during interactions with their children.^4^ In early childhood, parental technoference is associated with decreases in parent-child engagement,^5,6^ reduced ability to notice and attend to children’s needs,^7,8^ less frequent and lower-quality joint play and conversational turns,^9,10,11^ more negative responses to children’s behavior, and higher risk of child injury.^12^ In adolescence, adolescent-perceived parental technoference is associated with higher levels of parent-child conflict and lower levels of parental emotional support and warmth.^13,14^ When children’s emotional and physical needs are consistently ignored or inappropriately responded to, they are at risk of developing mental health difficulties,^15,16,17^ underscoring the need to investigate parental technoference as a potential precipitant of the development of mental health difficulties, such as depression, anxiety, hyperactivity, and inattention.

Existent research on parental technoference supports associations between parental technoference and child mental health difficulties across different developmental periods.^18,19^ An important limitation, however, is that this body of research has been primarily cross-sectional, which hinders our ability to understand the directionality of associations (ie, which comes first, parental technoference or child mental health difficulties?).^18,19,20,21^ A longitudinal study in children aged 1 to 5 years suggests that parent-rated technoference is associated with child anxiety and depression, while bidirectional associations exist between parental technoference and child attention problems and aggression.^21^ However, longitudinal designs are lacking at developmental periods other than early childhood, including the emerging adolescent years (ie, 9-12 years).^19^ This age range represents a sensitive period of brain development and is associated with an increased risk for mental health difficulties.^22,23,24,25^ During these years, parental responsiveness remains important for emerging adolescents’ well-being.^16^

While it is plausible that higher levels of parental technoference could precipitate emerging adolescents’ mental health difficulties, it is also possible that such difficulties exacerbate parent engagement in technoference. That is, parents of emerging adolescents with depression, anxiety, hyperactivity, or inattention symptoms may withdraw from interacting with their child over time and use technology to cope with stress related to their child’s mental health difficulties. Attaining clarity on the directionality of associations over time between perceptions of parental technoference and emerging adolescents’ mental health difficulties is critical to identifying the best targets for mental health prevention and intervention efforts for emerging adolescents.

This longitudinal study examined the bidirectional associations between emerging adolescents’ perceptions of parental technoference and their anxiety, depression, attention difficulties, and hyperactivity symptoms. Based on past research, bidirectional associations between perceptions of parental technoference and emerging adolescents’ mental health were expected, with potential differences in associations based on the type of mental health assessed. Given the lack of research distinguishing associations based on gender despite different mental illness onset and levels,^26,27^ the moderating role of gender was considered in an exploratory question.

## Methods

### Participants and Procedure

The present study uses the All Our Families cohort, a prospective pregnancy cohort study of maternal and child health determinants.^28^ Pregnant mothers were recruited from Calgary Laboratory Services, health care, and laboratory offices between May 3, 2008, and December 13, 2010, in Calgary, Alberta, Canada. Included mothers were (1) at least 18 years of age, (2) fluent in English, (3) recruited at a gestational age of at least 24 weeks, and (4) receiving community-based prenatal care. Of the women approached, 84% agreed to participate. Details about attrition across waves are reported elsewhere.^29^ The current secondary analysis uses data from the later waves of data collection that occurred during the COVID-19 pandemic. Families for which emerging adolescents participated in at least 1 of these waves were included in this study. Emerging adolescents who participated in at least 1 of those waves (n = 1303) were similar to those who did not (n = 665) with respect to family income (*t* = −1.03; mean [SD] scores, 6.23 [1.88] for participants vs 6.15 [1.70] for nonparticipants; 95% CI, −0.26 to 0.08]), depression (*t* = 0.75; mean [SD] scores, 50.05 [9.71] for participants vs 50.41 [9.52] for nonparticipants; 95% CI, −0.59 to 1.31]), anxiety (*t* = −0.18; mean [SD] scores, 49.30 [10.26] for participants vs 49.21 [9.83] for nonparticipants; 95% CI, −1.07 to 0.89]), or hyperactivity (*t* = 1.67; mean [SD] scores, 50.12 [9.61] for participants vs 50.93 [9.69] for nonparticipants; 95% CI, −0.15 to 1.77]) at the previous wave. There was some evidence, however, that participants had lower symptoms of attention difficulties than those who did not participate (*t* = 2.17; mean [SD] scores, 49.63 [9.30] for participants vs 50.63 [9.08] for nonparticipants; 95% CI, 0.10, 1.90).

Mothers were contacted to complete a series of online questionnaires 3 times during the COVID-19 pandemic and, at each time, were invited to provide written consent for their child to participate. Emerging adolescents provided written assent at each wave. The time 1 wave occurred between May 20 and July 15, 2020 (mean [SD] age, 9.7 [0.8] years); the time 2 wave, between March 4 and April 30, 2021 (mean [SD] age, 10.4 [0.9] years); and the time 3 wave, between November 22, 2021, and January 17, 2022 (mean [SD] age, 11.1 [0.9] years). This study was approved by the University of Calgary Institutional Ethics Board and adheres to the American Association for Public Opinion Research (AAPOR) reporting guidance for survey studies and the Strengthening the Reporting of Observational Studies in Epidemiology (STROBE) reporting guideline for cohort studies.

### Measures

Emerging adolescents’ perceptions of parental technoference were measured using a composite of 2 questions adapted from preexisting technoference measures^2^: “I wish my parent would spend less time on their phone and other devices,” and “I get frustrated with my parent for being on their phone or other devices when we’re spending time together.” Items were rated on a 4-point scale from “never” to “almost always.” The internal consistency between the 2 items was high at all time points (α > .78). This measure is consistent with other measures during this developmental period asking adolescents to report on parental technoference.^14,19,30,31,32^

Emerging adolescents’ reports of anxiety, depression, attention difficulties, and hyperactivity symptoms were assessed using the Behavior Assessment Scale for Children (BASC-3). Items are rated on a 4-point scale ranging from “never” to “almost always.” Standardized *t* scores were used. Higher scores indicated higher mental health difficulties. The BASC-3 is a commonly used measure of mental health in adolescence with strong psychometric properties.^33^ Scores were treated continuously in the present study given that low-risk samples have low proportions of participants with clinically significant scores (2%-10% in the present sample). Regarding gender, emerging adolescents also reported whether they identified as female, male, or another gender identity.

### Statistical Analysis

The random-intercept cross-lagged panel model (RI-CLPM) was used,^34,35^ which estimates traitlike differences between individuals (the random intercept), that is, differences between individuals that do not vary over time (eg, some people generally have more anxiety than others, which is considered a traitlike difference). The RI-CLPM analysis estimates between-family associations between variables (ie, the correlation between technoference and mental health difficulties; referred to as the between-family association) and calculates the within-family associations (controlling for the random intercept) to produce more accurate estimates of time-varying trajectories. The within-family associations include both cross-sectional associations (eg, do higher levels of technoference beyond the traitlike level translate into higher mental health difficulties?) along with cross-lags from one variable to the other (eg, are higher mental health difficulties at time 1 associated with higher technoference at time 2?), which are used to determine the directionality of associations. The RI-CLPMs allow us to control for traitlike factors that are not specifically measured in a given study.^36^ Additionally, given the reliance of the RI-CLPM on structural equation modeling principles, it is possible to test for gender differences using a multigroup approach.

Analyses were conducted in R, version 4.2.2^36^ using the psych^37^ and lavaan^38^ packages (R Program for Statistical Computing). Analyses were conducted using full information maximum likelihood to account for missing data and skewness. Descriptive statistics and correlational analyses are first reported, followed by the results of the RI-CLPMs for different types of mental health difficulties (ie, anxiety, depression, attention difficulties, and hyperactivity) given that different mechanisms may underlie each type. Model fit was assessed based on standard guidelines and cutoff criteria.^39^ Values superior to 0.95 were deemed adequate for the comparative fit index and the Tucker-Lewis Index. For the root mean square error of approximation and the standardized root mean squared residual, values greater than 0.06 were deemed adequate. A traditional CLPM was first tested to ensure that the RI-CLPM presented a better fit using a χ^2^ difference test. Autoregressive paths were then constrained to see if this model was a better fit than the regular unconstrained RI-CLPM compared again with the χ^2^ difference test. The gender comparison was conducted using a multigroup analysis.^40^ The number of gender-diverse participants (n = 8) was insufficient for a multigroup comparison; the gender comparison thus focused on those who identified as boys or girls. Whether or not 95% CIs between boys and girls overlapped was examined to determine possible different associations across genders. This study did not rely on statistical significance, but instead on the magnitude of effect sizes to determine meaningful effects. The magnitudes of effect sizes were interpreted using standard guidelines,^41^ with 0.10 representing small, 0.20 representing moderate, and 0.30 representing large effect sizes. Data were analyzed from December 1 to 31, 2023.

## Results

Participants included 1303 emerging adolescents (529 of 1028 [51.5%] identified as girls, 491 [47.8%] identified as boys, and 8 [0.8%] identified as gender diverse [eg, transgender, gender fluid, agender], and the remaining 275 did not report this information) with a mean (SD) age of 9.7 (0.8) years at time 1 (see Table 1). Table 2 presents the descriptive statistics and correlations between study variables. For the main analysis, models were fitted for each type of mental health difficulty. For anxiety, attention difficulties, and hyperactivity, the best-fitting model was the standard RI-CLPM model (ie, without any constraints on autoregressive paths) (eTable 1 in Supplement 1). Depression could not be estimated using the RI-CLPM model given negative variances for the overall model and errors, indicating that the model could not be successfully estimated with the parameters of the model; however, the model by gender was successfully estimated, and these findings are reported in eTables 2 and 3 in Supplement 1.

**Table 1.** Participant Sociodemographic Information

Characteristic	Data
Child gender, No. (%)^a^	
Female	529 (51.5)
Male	491 (47.8)
Other	8 (0.8)
Child age, mean (SD), y	
Time 1	9.7 (0.8)
Time 2	10.4 (0.9)
Time 3	11.1 (0.9)
Maternal age, mean (SD), y	
Time 1	41.5 (4.4)
Time 2	42.3 (4.4)
Time 3	43.0 (4.4)
Family yearly income (time 1), No. (%)^b^	
<$40 000	44 (4.2)
$40 000-$80 000	116 (11.0)
>$80 000	896 (84.8)

^a^ Includes 1028 respondents.

^b^ Includes 1056 respondents. The 3 categories represented low, middle, and high incomes in the recruitment location at the time of the study and are expressed in Canadian dollars.

**Table 2.** Descriptive Statistics and Correlations Between Study Variables

	Technoference			Anxiety			Depression			Attention difficulty			Hyperactivity		
	T1	T2	T3	T1	T2	T3	T1	T2	T3	T1	T2	T3	T1	T2	T3
**Correlations (95% CI)**															
Technoference T1	NA	NA	NA	NA	NA	NA	NA	NA	NA	NA	NA	NA	NA	NA	NA
Technoference T2	0.53 (0.47-0.58)	NA	NA	NA	NA	NA	NA	NA	NA	NA	NA	NA	NA	NA	NA
Technoference T3	0.43 (0.37-0.49	0.60 (0.55-0.64)	NA	NA	NA	NA	NA	NA	NA	NA	NA	NA	NA	NA	NA
Anxiety T1	0.22 (0.15-0.28)	0.19 (0.12-0.26)	0.18 (0.11-0.25)	NA	NA	NA	NA	NA	NA	NA	NA	NA	NA	NA	NA
Anxiety T2	0.16 (0.08-0.23)	0.28 (0.22-0.34)	0.24 (0.18-0.31)	0.67 (0.63-0.71)	NA	NA	NA	NA	NA	NA	NA	NA	NA	NA	NA
Anxiety T3	0.10 (0.02-0.17)	0.18 (0.12-0.25)	0.29 (0.23-0.34)	0.55 (0.50-0.6)	0.68 (0.64-0.71)	NA	NA	NA	NA	NA	NA	NA	NA	NA	NA
Depression T1	0.32 (0.26-0.38)	0.21 (0.14-0.28)	0.16 (0.09-0.23)	0.67 (0.63-0.71)	0.41 (0.34-0.46)	0.37 (0.30-0.43)	NA	NA	NA	NA	NA	NA	NA	NA	NA
Depression T2	0.22 (0.15-0.29)	0.34 (0.28-0.39)	0.26 (0.19-0.32)	0.52 (0.46-0.57)	0.69 (0.66-0.72)	0.53 (0.48-0.58)	0.55 (0.50-0.60)	NA	NA	NA	NA	NA	NA	NA	NA
Depression T3	0.13 (0.05-0.20)	0.16 (0.09-0.23)	0.27 (0.21-0.32)	0.40 (0.34-0.46)	0.45 (0.40-0.51)	0.74 (0.71-0.76)	0.43 (0.37-0.49)	0.61 (0.56-0.65)	NA	NA	NA	NA	NA	NA	NA
Attention difficulty T1	0.12 (0.05-0.18)	0.12 (0.04-0.19)	0.12 (0.05-0.19)	0.42 (0.36-0.47)	0.26 (0.19-0.33)	0.21 (0.14-0.28)	0.40 (0.35-0.46)	0.27 (0.20-0.34)	0.22 (0.15-0.29)	NA	NA	NA	NA	NA	NA
Attention difficulty T2	0.09 (0.02-0.16)	0.19 (0.13-0.24)	0.16 (0.09-0.22)	0.36 (0.30-0.43)	0.42 (0.37-0.47)	0.30 (0.24-0.36)	0.33 (0.26-0.39)	0.42 (0.37-0.47)	0.30 (0.23-0.36)	0.68 (0.64-0.72)	NA	NA	NA	NA	NA
Attention difficulty T3	0.08 (0.01-0.16)	0.17 (0.10-0.24)	0.21 (0.15-0.27)	0.38 (0.32-0.44)	0.36 (0.30-0.42)	0.48 (0.43-0.52)	0.34 (0.28-0.41)	0.37 (0.31-0.43)	0.47 (0.42-0.52)	0.61 (0.56-0.65)	0.67 (0.64-0.71)	NA	NA	NA	NA
Hyperactivity T1	0.14 (0.07-0.20)	0.10 (0.03-0.18)	0.12 (0.05-0.19)	0.43 (0.38-0.48)	0.23 (0.16-0.3)	0.22 (0.15-0.29)	0.36 (0.30-0.42)	0.20 (0.13-0.27)	0.16 (0.09-0.23)	0.72 (0.69-0.75)	0.56 (0.51-0.61)	0.54 (0.48-0.59)	NA	NA	NA
Hyperactivity T2	0.11 (0.04-0.19)	0.22 (0.16-0.28)	0.18 (0.12-0.25)	0.34 (0.28-0.41)	0.39 (0.34-0.44)	0.28 (0.22-0.34)	0.25 (0.18-0.32)	0.31 (0.25-0.36)	0.21 (0.15-0.28)	0.56 (0.51-0.61)	0.69 (0.66-0.72)	0.55 (0.50-0.60)	0.66 (0.62-0.70)	NA	NA
Hyperactivity T3	0.07 (-0.01-0.14)	0.15 (0.09-0.22)	0.23 (0.18-0.29)	0.33 (0.26-0.39)	0.32 (0.26-0.38)	0.46 (0.41-0.51)	0.24 (0.17-0.31)	0.26 (0.19-0.32)	0.38 (0.33-0.44)	0.50 (0.44-0.55)	0.54 (0.49-0.59)	0.74 (0.71-0.76)	0.59 (0.54-0.64)	0.67 (0.63-0.70)	NA
**Descriptive statistics**															
Mean (SD) BASC-3 [range] score^a^	3.6 (1.5) [2-8]	3.6 (1.5) [2-8]	3.5 (1.5) [2-8]	49.4 (10.8) [33-89]	49.0 (11.5) [33-89]	50.2 (12.7) [33-91]	49.4 (9.6) [40-98]	49.5 (11.0) [40-103]	51.3 (13.1) [40-103]	49.1 (10.3) [35-81]	48.7 (10.6) [35-83]	50.0 (11.7) [35-81]	47.9 (9.6) [34-84]	48.0 (9.7) [34-84]	48.8 (10.5) [34-89]
No. (%) of participants above clinical cutoff	NA	NA	NA	51 (5.8)	72 (7.1)	98 (9.6)	46 (5.2)	73 (7.2)	101 (9.9)	33 (3.7)	55 (5.4)	86 (8.4)	20 (2.2)	28 (2.7)	45 (4.4)
No. of participants	881	1025	1020	884	1019	1022	884	1020	1022	884	1020	1023	884	1021	1023

Abbreviations: BASC-3, Behavior Assessment Scale for Children; NA, not applicable; T1, time 1 (9 years of age); T2, time 2 (10 years of age); T3, time 3 (11 years of age).

^a^ Clinically, scores below 30 are considered very low; 31 to 40, low; 41 to 59, average; 60 to 69, at risk; and 70 and above, clinically significant.

Second, between- and within-family associations between perceptions of parental technoference and emerging adolescents’ mental health difficulties were examined. The between-family (time-invariant) portion of the RI-CLPMs indicated moderate correlations between the random intercepts, with large variations in the 95% CIs (Table 3 and Figure). This indicates that, in general, when emerging adolescents perceived their parent to engage in more technoference, they presented higher mental health difficulties (*r* range, 0.17-0.19). The within-family (time-varying) cross-sectional correlations were most consistent for perceived parental technoference and anxiety, for which correlations were moderate in magnitude at all study times (*r* range, 0.21-0.28). The correlations between perceived parental technoference and attention difficulties and hyperactivity ranged from small to large depending on the time point (*r* range, 0.06-0.27) (Table 2 and Figure).

**Table 3.** Parameters for RI-CLPMs

Association	Effect size, β (95% CI)^a^		
	Anxiety	Attention difficulties	Hyperactivity
Unstandardized random-intercept variance			
Technoference	0.82 (0.58 to 1.06)	0.84 (0.61 to 1.07)	0.83 (0.60 to 1.07)
MH	65.83 (48.81 to 82.54)	73.12 (62.64 to 83.60)	58.25 (49.57 to 66.92)
Within-family autoregressive paths^b^			
Technoference T1 → technoference T2	0.22 (0.08 to 0.36)	0.34 (0.22 to 0.47)	0.24 (0.10 to 0.38)
Technoference T2 → technoference T3	0.32 (0.20 to 0.45)	0.24 (0.10 to 0.38)	0.33 (0.20 to 0.46)
MH T1 → MH T2	0.28 (0.06 to 0.50)	0.06 (−0.18 to 0.30)	0.09 (−0.11 to 0.29)
MH T2 → MH T3	0.40 (0.27 to 0.53)	0.19 (0.04 to 0.33)	0.17 (0.02 to 0.33)
Between-family associations^b^			
Technoference ↔ MH	0.25 (−0.12 to 0.63)	0.19 (0.06 to 0.33)	0.17 (0.03 to 0.31)
Within-family cross-sectional associations^b^			
Technoference T1 ↔ MH T1	0.21 (0.05 to 0.37)	0.06 (−0.10 to 0.22)	0.13 (−0.02 to 0.27)
Technoference T2 ↔ MH T2	0.28 (0.17 to 0.39)	0.20 (0.05 to 0.35)	0.27 (0.13 to 0.41)
Technoference T3 ↔ MH T3	0.25 (0.17 to 0.32)	0.17 (0.09 to 0.26)	0.24 (0.15 to 0.32)
Within-family cross-lagged associations^b^			
Technoference T1 → MH T2	0.03 (−0.11 to 0.17)	0.00 (−0.16 to 0.16)	0.07 (−0.07 to 0.22)
Technoference T2 → MH T3	0.03 (−0.07 to 0.12)	0.12 (0.001 to 0.24)	0.11 (−0.02 to 0.24)
MH T1 → technoference T2	0.11 (−0.05 to 0.26)	0.04 (−0.13 to 0.20)	0.01 (−0.14 to 0.16)
MH T2 → technoference T3	0.12 (0.001 to 0.24)	0.05 (−0.07 to 0.16)	0.08 (−0.03 to 0.20)

Abbreviations: MH, mental health; RI-CLPMs, random-intercepts cross-lagged panel models; arrows, directionality.

^a^ The depression model could not be estimated due to negative variances in the RI-CLPM and errors.

^b^ Expressed as standardized values.

**Figure.**
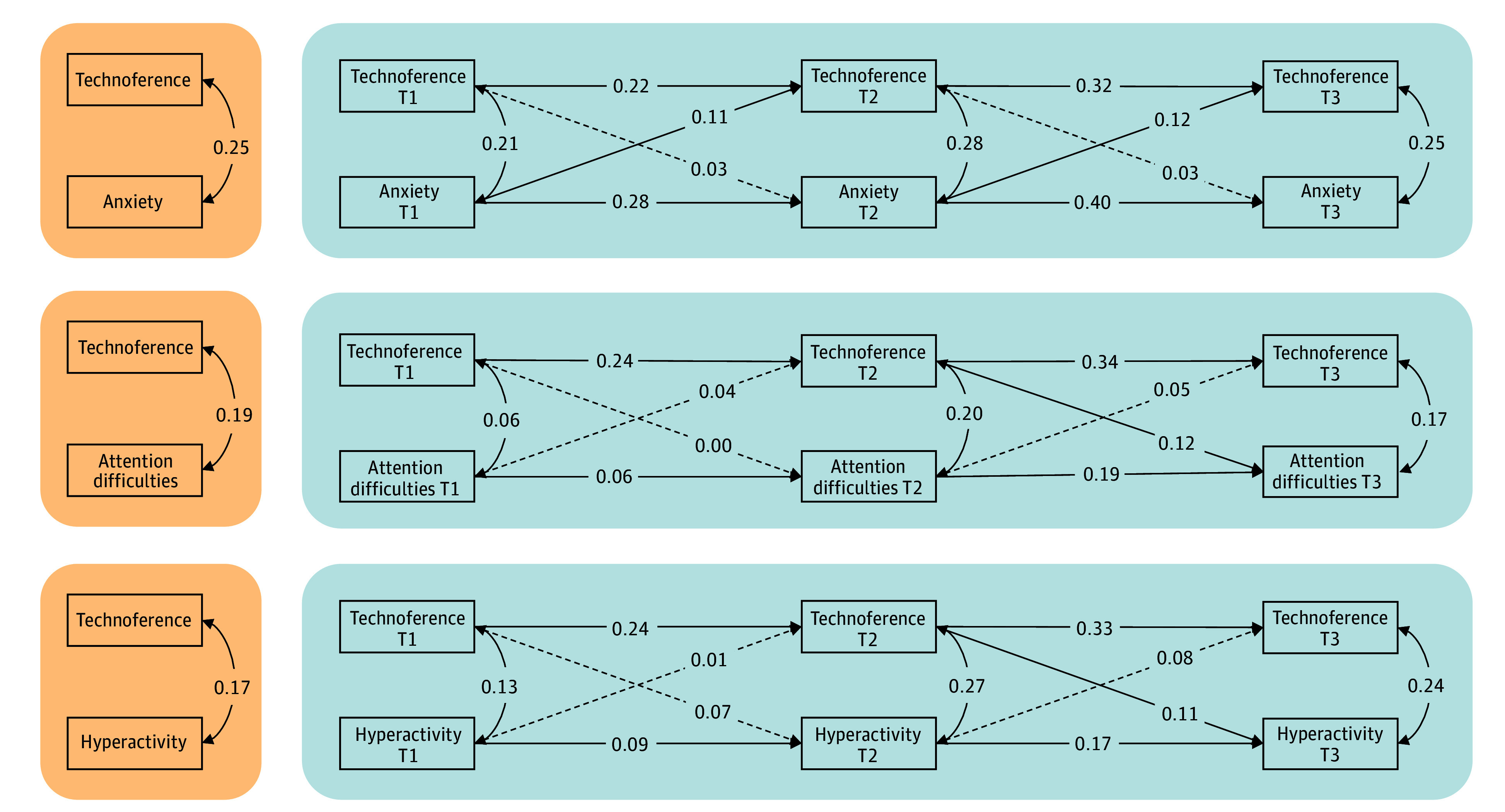
Graphical Depiction of Standardized Estimates for the Random-Intercepts Cross-Lagged Panel Models of Anxiety, Attention Difficulties, and Hyperactivity Effect sizes of 0.10 represent small effect sizes; 0.20, moderate effect sizes; and 0.30, large effect sizes. Solid lines represent parameter estimates with a magnitude in excess of the threshold representing a small effect size (ie, ≥0.10). Dashed lines represent parameter estimates with a magnitude that was less than the threshold representing a small effect size. Orange indicates between components; blue, within components. Descriptive statistics and correlations between study variables are presented in Table 2. T1 indicates time 1 (9 years of age); T2, time 2 (10 years of age); and T3, time 3 (11 years of age).

The within-family cross-lag associations varied based on the type of mental health difficulties assessed (Table 3 and Figure). Associations from higher levels of anxiety to higher levels of perceived parental technoference were observed at 10 (β = 0.11 [95% CI, −0.05 to 0.26]) and 11 (β = 0.12 [95% CI, 0.001-0.24]) years of age (small effect sizes), while the reverse was not true. For attention difficulties and hyperactivity, there was some evidence for directionality of an association from perceived parental technoference to emerging adolescents’ mental health difficulties (β = 0.07 [95% CI, −0.07 to 0.22]), although mostly from time 2 to time 3 (hyperactivity, β = 0.11 [95% CI, −0.02 to 0.24]; inattention, β = 0.12 [95% CI, 0.001-0.24]). Small effect sizes were identified for associations from mental health difficulties to perceived parental technoference.

Last, gender differences were examined for the associations between perceptions of parental technoference and emerging adolescents’ mental health. The subgroup analysis revealed some associations of different magnitudes between girls and boys as depicted in eTables 2 to 5 in Supplement 1. However, all 95% CIs overlapped and did not suggest meaningful differences.

## Discussion

Technoference is becoming a pervasive part of parent-child interactions,^2,3^ and a growing literature suggests that technology-based interferences have mental health ramifications.^18,19^ This literature is, however, mostly based on cross-sectional studies, which limits our understanding of the directionality of associations between parental technoference and emerging adolescents’ outcomes. The present cohort study leveraged longitudinal data to explicitly test the bidirectional associations between parental technoference and emerging adolescents’ mental health using robust methods that more closely approximate causality in longitudinal studies (ie, RI-CLPM). Higher levels of emerging adolescent anxiety symptoms were associated with higher levels of perceived parental technoference (but not vice versa). Higher levels of perceived parental technoference were associated with higher levels of emerging adolescent inattention and hyperactivity symptoms (but not vice versa). Substantial gender differences were not identified. It is possible that, despite experiencing different levels and onset of mental health difficulties,^26,27^ boys and girls similarly experience the effects of parental technoference.

The between-family and cross-sectional within-family levels of the RI-CLPMs replicated past cross-sectional findings showing that higher perceived parental technoference was associated with higher mental health difficulties in emerging adolescents, particularly symptoms of anxiety.^2^ Leveraging the strength of the within-family cross-lags, different directionalities were identified depending on the type of mental health difficulties investigated. Specifically, there was evidence of unidirectional associations from emerging adolescents’ anxiety to later perceived parental technoference but not from perceived parental technoference to later anxiety. By contrast, the cross-lags showed associations from higher perceived parental technoference to more inattention and hyperactivity in emerging adolescents at later study times.

While not measured directly in this study, parents seeing their emerging adolescent struggling with anxiety may be more likely to reach out for their technological devices as an escape from difficult and tense interactions.^42^ Their concern about their child’s anxiety may lead parents to reach out to family, friends, or other caregivers digitally for support or prompt them to access online information, forums, or social media, with the goal of getting further support in the face of their child’s difficulties.^43^ Emerging adolescents with anxiety may also be more sensitive to their parents’ technoference than emerging adolescents with lower levels of anxiety, resulting in higher perceptions of parental technoference. People with anxiety are theorized to have information processing biases that underlie or maintain their anxiety symptoms and may change their perceptions of interactions.^44,45^

In contrast, perceived parental technoference was associated with higher later attention difficulties and hyperactivity in emerging adolescents (with only limited evidence of the reverse association). This finding is consistent with past longitudinal findings identifying associations between parental technoference and attention problems and aggression in early childhood,^21^ suggesting that children’s and adolescents’ environment can exacerbate neurodevelopmental symptoms. Interestingly, explanations for this association typically focus on behavioral escalation in children (eg, children acting up to gain their parents’ attention).^6^ In the present study, however, small cross-lag associations with attention difficulties were also identified. Future studies should examine whether parent technoference may prompt emerging adolescents to also reach out for their own devices, which may in turn foster further attention difficulties.^45^

Future research should seek to advance our understanding of the direction of associations from emerging adolescents’ mental health to parental technoference and their underlying mechanisms. This requires multimethod longitudinal studies with different age groups to determine whether the pattern of findings observed herein cross stages of development.^46^ Additionally, future studies should examine the specific activities in which parents are engaged during instances of technoference, as well as their degree of digital absorption. Given that parental technoference can lead to higher risk for emerging adolescents’ attention difficulties and hyperactivity, it is critical that discussions of screen use and technoference become a family affair within health care contexts. Motivation is a key ingredient for behavioral change, and information about the link between parental technoference and emerging adolescents’ mental health difficulties could be the impetus for parents reducing their phone use and, as a result, their technoference habits.

Last, a more systematic investigation of risk factors for parental technoference would be beneficial to preemptively identify parents who engage regularly in technoference, which can in turn provide insights into target prevention efforts. Research has shown that parents who have more supportive attitudes toward technoference (eg, believe that using technology to cope is not problematic) engage in more technology use.^47,48^ In cross-sectional studies, parents with high levels of technoference report lower well-being,^49^ and in longitudinal studies, parents with high levels of stress engage in more technoference over time.^20^ Importantly, parents consider their phone use to be something that is modifiable, but they perceive a low locus of control for this change.^50^ Targeted and supportive efforts may be key to helping parents limit their technology use during interactions with their child.

### Limitations

This study relied on a single-informant design such that emerging adolescents reported on all variables. This design has its advantages given that adolescents are considered the best informant of their mental health.^51^ However, an emerging adolescent who is struggling with anxiety may perceive their parents as engaging more with their technological device during interactions. Future research should include multiple informants and/or observational assessments of technoference. The study also provided little information for depression specifically given that the model could not be identified. Participants included in the All Our Families cohort were primarily White and had a middle to high income status, which is representative of the geographical region, but potentially prevents generalizability to rural or more vulnerable communities. Future research should seek to expand technoference research beyond White, educated, industrialized, rich, and democratic (so-called WEIRD) populations. Last, the data collection took place over the course of the COVID-19 pandemic, a time where the everyday life of families (including technology use) drastically changed.^52,53^ This period may not reflect the regular daily life of families given that families had to cope with increased stressors and added household responsibilities (eg, assisting with children’s at-home school learning). Nonetheless, the increased stress and work-from-home arrangements for many parents may have contributed to increased screen time,^54,55^ providing a natural experiment to test the directionality of associations.

## Conclusions

In this 3-wave longitudinal cohort study of parental technoference and emerging adolescent mental health, perceived parental technoference was associated with current and prospective mental health symptoms during emerging adolescence. The findings contribute to a burgeoning literature on the association between parental technoference and child outcomes, which has not been explored with robust methods, such as RI-CLPMs. This study highlights the complex relations between parental technoference and emerging adolescents’ mental health and highlights the need to address parental technology use when considering emerging adolescents’ well-being.

## Notes

Supplement 1. **eTable 1.** Model Fit and χ^2^ Difference Tests **eTable 2.** Unstandardized Parameters for the RI-CLPM Anxiety Model With Subgroup Analysis by Gender **eTable 3.** Unstandardized Parameters for the RI-CLPM Depression Model With Subgroup Analysis by Gender **eTable 4.** Unstandardized Parameters for the RI-CLPM Attention Difficulties Model With Subgroup Analysis by Gender **eTable 5.** Unstandardized Parameters for the RI-CLPM Hyperactivity Model With Subgroup Analysis by Gender

Supplement 2.
Data Sharing Statement
