# Supplementary material for: Perceived Parental Distraction by Technology and Mental Health Among Emerging Adolescents
Source: JAMA Netw Open. 2024 Aug 16;7(8):e2428261. doi: 10.1001/jamanetworkopen.2024.28261 (PMC11329881; doi:10.1001/jamanetworkopen.2024.28261)
Supplement: Supplement 1. — eTable 1. Model Fit and χ2 Difference Tests eTable 2. Unstandardized Parameters for the RI-CLPM Anxiety Model With Subgroup Analysis by Gender eTable 3. Unstandardized Parameters for the RI-CLPM Depression Model With Subgroup Analysis by Gender eTable 4. Unstandardized Parameters for the RI-CLPM Attention Difficulties Model With Subgroup Analysis by Gender eTable 5. Unstandardized Parameters for the RI-CLPM Hyperactivity Model With Subgroup Analysis by Gender [file jamanetwopen-e2428261-s001.pdf]

Supplementary Online Content

Deneault AA, Plamondon A, Neville RD, et al. Perceived parental technoference and mental health among emerging adolescents. *JAMA Netw Open*. 2024;7(8):e2428261. doi:10.1001/jamanetworkopen.2024.28261

eTable 1. Model Fit and χ^2 Difference Tests
eTable 2. Unstandardized Parameters for the RI-CLPM Anxiety Model With Subgroup Analysis by Gender
eTable 3. Unstandardized Parameters for the RI-CLPM Depression Model With Subgroup Analysis by Gender
eTable 4. Unstandardized Parameters for the RI-CLPM Attention Difficulties Model With Subgroup Analysis by Gender
eTable 5. Unstandardized Parameters for the RI-CLPM Hyperactivity Model With Subgroup Analysis by Gender

This supplementary material has been provided by the authors to give readers additional information about their work.

© 2024 Deneault AA et al. *JAMA Network Open*.

eTable 1. Model Fit and χ2 Difference Tests

	RMSEA	SRMR	CFI	TLI	χ^2	*df*	*p*	$\Delta\chi^2$	*df*	*p*
Parental technoference ←→ Anxiety										
RI-CLPM (*final fitted model*)	.03	.01	.999	.991	2.15	1	.142	–	–	–
RI-CLPM (*constrained*)	.02	.02	.998	.995	7.84	5	.166	5.68	4	.224
CLPM (*traditional*)	.09	.03	.977	.913	46.35	4	< .001	44.20	3	< .001
Parental technoference ←→ Depression										
RI-CLPM (*final fitted model*)[a]	–	–	–	–	–	–	–	–	–	–
RI-CLPM (*constrained*)	.07	.05	.975	.947	45.89	7	< .001	–	–	–
CLPM (*traditional*)	.08	.04	.966	.914	60.35	6	< .001	–	–	–
Parental technoference ←→ Attention difficulties										
RI-CLPM (*final fitted model*)	.01	.01	1.00	.998	1.19	1	.275	–	–	–
RI-CLPM (*constrained*)	.03	.02	.997	.992	9.60	5	.088	8.41	4	.078
CLPM (*traditional*)	.12	.04	.958	.843	77.49	4	< .001	76.30	3	< .001
Parental technoference ←→ Hyperactivity										
RI-CLPM (*final fitted model*)	.03	.01	.999	.991	2.08	1	.149	–	–	–
RI-CLPM (*constrained*)	.02	.02	.999	.997	6.84	5	.233	4.75	4	.314
CLPM (*traditional*)	.12	.04	.957	.840	78.71	4	< .001	76.63	3	< .001

Note. [a] This model could not be estimated due to negative variances.

© 2024 Deneault AA et al. *JAMA Network Open*.

eTable 2. Unstandardized Parameters for the RI-CLPM Anxiety Model With Subgroup Analysis by Gender

Associations	Girls		Boys	
	ES	95% CI	ES	95% CI
**Unstandardized random intercept variance**				
Tech	0.71	[0.27, 1.15]	0.83	[0.55, 1.22]
MH	65.62	[26.74, 104.51]	60.93	[41.99, 79.87]
**Within-person autoregressive paths**[a]				
Tech $_{T1}$ → Tech $_{T2}$	0.24	[0.01, 0.48]	0.22	[0.03, 0.41]
Tech $_{T2}$ → Tech $_{T3}$	0.35	[0.17, 0.54]	0.30	[0.12, 0.50]
MH $_{T1}$ → MH $_{T2}$	0.49	[0.05, 0.93]	0.26	[0.03, 0.50]
MH $_{T2}$ → MH $_{T3}$	0.61	[0.42, 0.80]	0.32	[0.09, 0.56]
**Between-person association**[a]				
Tech ↔ MH	0.25	[-0.12, 0.63]	0.30	[0.08, 0.52]
**Within-person cross-sectional associations**[a]				
Tech $_{T1}$ ↔ MH $_{T1}$	0.34	[0.06, 0.62]	0.13	[-0.06, 0.33]
Tech $_{T2}$ ↔ MH $_{T2}$	0.26	[0.11, 0.40]	0.29	[0.13, 0.46]
Tech $_{T3}$ ↔ MH $_{T3}$	0.35	[0.24, 0.46]	0.21	[0.08, 0.34]
**Within-person cross-lagged effects**[a]				
Tech driven effects				
Tech $_{T1}$ → MH $_{T2}$	-0.03	[-0.22, 0.16]	0.06	[-0.12, 0.24]
Tech $_{T2}$ → MH $_{T3}$	-0.01	[-0.13, 0.12]	0.08	[-0.09, 0.25]
MH driven effects				
MH $_{T1}$ → tech $_{T2}$	0.38	[-0.00, 0.76]	0.27	[0.04, 0.50]
MH $_{T2}$ → tech $_{T3}$	0.50	[0.32, 0.68]	0.31	[0.08, 0.54]

Note. CI = confidence interval; ES = Effect sizes; MH = mental health; Tech = technoference. [a] Standardized values.

© 2024 Deneault AA et al. *JAMA Network Open*.

eTable 3. Unstandardized Parameters for the RI-CLPM Depression Model With Subgroup Analysis by Gender

**Associations**	**Girls**		**Boys**	
	**ES**	**95% CI**	**ES**	**95% CI**
**Unstandardized random intercept variance**				
Tech	0.59	[0.19, 0.99]	0.92	[0.63, 1.21]
MH	54.81	[37.44, 72.17]	49.28	[16.65, 81.91]
**Within-person autoregressive paths**[a]				
$Tech_{T1} \rightarrow Tech_{T2}$	0.33	[0.10, 0.57]	0.20	[0.03, 0.37]
$Tech_{T2} \rightarrow Tech_{T3}$	0.41	[0.25, 0.56]	0.25	[0.07, 0.42]
$MH_{T1} \rightarrow MH_{T2}$	-0.22	[-0.68, 0.23]	0.24	[-0.16, 0.64]
$MH_{T2} \rightarrow MH_{T3}$	0.38	[0.26, 0.51]	0.45	[0.24, 0.67]
**Between-person association**[b]				
Tech ↔ MH	0.32	[0.01, 0.62]	0.27	[0.00, 0.53]
**Within-person cross-sectional associations**[b]				
$Tech_{T1} \leftrightarrow MH_{T1}$	0.53	[0.30, 0.76]	0.28	[0.07, 0.48]
$Tech_{T2} \leftrightarrow MH_{T2}$	0.29	[0.09, 0.50]	0.40	[0.24, 0.56]
$Tech_{T3} \leftrightarrow MH_{T3}$	0.30	[0.21, 0.39]	0.21	[0.09, 0.33]
**Within-person cross-lagged effects**[a]				
Tech driven effects				
$Tech_{T1} \rightarrow MH_{T2}$	0.37	[-0.01, 0.74]	0.10	[-0.09, 0.30]
$Tech_{T2} \rightarrow MH_{T3}$	-0.05	[-0.18, 0.09]	-0.05	[-0.17, 0.08]
MH driven effects				
$MH_{T1} \rightarrow tech_{T2}$	0.02	[-0.25, 0.29]	0.07	[-0.14, 0.28]
$MH_{T2} \rightarrow tech_{T3}$	0.14	[0.01, 0.27]	0.12	[-0.08, 0.31]

Note. CI = confidence interval; ES = Effect sizes; MH = mental health; Tech = technoference. [a] Standardized values.

© 2024 Deneault AA et al. *JAMA Network Open*.

eTable 4. Unstandardized Parameters for the RI-CLPM Attention Difficulties Model With Subgroup Analysis by Gender

Associations	Girls		Boys	
	ES	95% CI	ES	95% CI
**Unstandardized random intercept variance**				
Tech	0.67	[0.26, 1.09]	0.92	[0.58, 1.25]
MH	67.59	[51.71, 83.47]	84.17	[68.03, 100.30]
**Within-person autoregressive paths**[a]				
Tech $_{T1}$ → Tech $_{T2}$	0.31	[0.08, 0.53]	0.21	[0.02, 0.40]
Tech $_{T2}$ → Tech $_{T3}$	0.42	[0.24, 0.60]	0.28	[0.10, 0.47]
MH $_{T1}$ → MH $_{T2}$	-0.12	[-0.62, 0.37]	0.08	[-0.22, 0.38]
MH $_{T2}$ → MH $_{T3}$	0.24	[0.06, 0.43]	0.06	[-0.19, 0.32]
**Between-person association**[b]				
Tech ↔ MH	0.39	[0.14, 0.63]	0.14	[-0.05, 0.32]
**Within-person cross-sectional associations**[b]				
Tech $_{T1}$ ↔ MH $_{T1}$	0.10	[-0.19, 0.39]	0.06	[-0.14, 0.27]
Tech $_{T2}$ ↔ MH $_{T2}$	0.09	[-0.18, 0.35]	0.25	[0.03, 0.48]
Tech $_{T3}$ ↔ MH $_{T3}$	0.26	[0.15, 0.38]	0.14	[-0.01, 0.28]
**Within-person cross-lagged effects**[a]				
Tech driven effects				
Tech $_{T1}$ → MH $_{T2}$	-0.03	[-0.32, 0.26]	0.09	[-0.12, 0.30]
Tech $_{T2}$ → MH $_{T3}$	0.00	[-0.16, 0.16]	0.25	[0.04, 0.46]
MH driven effects				
MH $_{T1}$ → tech $_{T2}$	-0.06	[-0.34, 0.21]	0.06	[-0.16, 0.29]
MH $_{T2}$ → tech $_{T3}$	0.03	[-0.11, 0.17]	0.06	[-0.11, 0.24]

Note. CI = confidence interval; ES = Effect sizes; MH = mental health; Tech = technoference. [a] Standardized values.

© 2024 Deneault AA et al. *JAMA Network Open*.

eTable 5. Unstandardized Parameters for the RI-CLPM Hyperactivity Model With Subgroup Analysis by Gender

Associations	Girls		Boys	
	ES	95% CI	ES	95% CI
**Unstandardized random intercept variance**				
Tech	0.67	[0.26, 1.08]	0.88	[0.54, 1.22]
MH	57.96	[45.94, 69.98]	58.13	[43.55, 72.70]
**Within-person autoregressive paths[a]**				
Tech $_{T1}$ → Tech $_{T2}$	0.32	[0.10, 0.54]	0.22	[0.04, 0.41]
Tech $_{T2}$ → Tech $_{T3}$	0.42	[0.23, 0.60]	0.25	[0.05, 0.44]
MH $_{T1}$ → MH $_{T2}$	0.03	[-0.29, 0.36]	0.17	[-0.08, 0.42]
MH $_{T2}$ → MH $_{T3}$	0.14	[-0.09, 0.38]	0.13	[-0.11, 0.38]
**Between-person association[b]**				
Tech ↔ MH	0.50	[0.24, 0.75]	0.08	[-0.15, 0.30]
**Within-person cross-sectional associations[b]**				
Tech $_{T1}$ ↔ MH $_{T1}$	0.09	[-0.14, 0.32]	0.13	[-0.07, 0.33]
Tech $_{T2}$ ↔ MH $_{T2}$	-0.02	[-0.28, 0.24]	0.39	[0.21, 0.57]
Tech $_{T3}$ ↔ MH $_{T3}$	0.25	[0.12, 0.37]	0.24	[0.10, 0.39]
**Within-person cross-lagged effects[a]**				
Tech driven effects				
Tech $_{T1}$ → MH $_{T2}$	-0.11	[-0.35, 0.13]	0.16	[-0.03, 0.34]
Tech $_{T2}$ → MH $_{T3}$	-0.07	[-0.27, 0.13]	0.20	[-0.01, 0.40]
MH driven effects				
MH $_{T1}$ → tech $_{T2}$	-0.17	[-0.40, 0.06]	0.10	[-0.10, 0.30]
MH $_{T2}$ → tech $_{T3}$	-0.03	[-0.19, 0.12]	0.17	[-0.02, 0.36]

Note. CI = confidence interval; ES = Effect sizes; MH = mental health; Tech = technoference. [a] Standardized values.

© 2024 Deneault AA et al. *JAMA Network Open*.
