# Supplementary material for: Perceived Parental Distraction by Technology and Mental Health Among Emerging Adolescents
Source: JAMA Netw Open. 2024 Aug 16;7(8):e2428261. doi: 10.1001/jamanetworkopen.2024.28261 (PMC11329881; doi:10.1001/jamanetworkopen.2024.28261)
Supplement: Supplement 2. — Data Sharing Statement [file jamanetwopen-e2428261-s002.pdf]

Data Sharing Statement

Deneault. Perceived Parental Technoference and Mental Health Among Emerging Adolescents. *JAMA Netw Open*. Published August 16, 2024.
doi:10.1001/jamanetworkopen.2024.28261

Data
Data available: Yes
Data types: Data dictionary
How to access data: Request to corresponding author.
When available: With publication

Supporting Documents
Document types: Statistical/analytic code
How to access documents: Request to corresponding author.
When available: With publication

Additional Information
Who can access the data: Researchers whose proposed use of the data has been approved.
Types of analyses: For approved purposes.
Mechanisms of data availability: After approval from the AOF process.
Any additional restrictions: The All Our Families Cohort data are available through requests to the study (https://ucalgary.ca/allourfamilies), and metadata of variables can be found at https://www.maelstrom-research.org/study/aof. Restrictions apply to the availability of these deidentified data, which were used according to data sharing agreements.
